# “Keep joking”: appreciation and production of humor in aging

**DOI:** 10.3389/fpsyg.2026.1754264

**Published:** 2026-05-05

**Authors:** Lucie Hevin, Yannick Gounden, Maéva Chignier, Aymeric Parant, Harmony Duclos

**Affiliations:** CRP-CPO, UR UPJV 7273, Université de Picardie Jules Verne, Amiens, France

**Keywords:** aging, humor forms, humor processing, positivity bias, social cognition

## Abstract

Humor is a universal human experience, enjoyed and expressed across all stages of life. While numerous studies have explored the developmental aspects of humor, relatively little research has focused on humor in older adults. Yet, humor remains a valuable psychological and social resource, aiding in stress management and fostering meaningful social connections. This study aims to explore individual differences in the appreciation and production of humor among older adults. A self-assessment questionnaire targeting the production and appreciation of six forms of humor (irony, puns, parody, dark humor, slapstick humor and satire) was administered to 37 young people (19–34 years old), 27 middle-aged people (40–59 years) and 37 elderly people (61–83 years old). Results showed that younger adults scored higher than older adults in both humor appreciation and production (all *p* < 0.001). Despite these age-related differences, the relative preferences for the six humor forms remained stable across groups. Correlational analyses conducted across the full sample revealed positive associations between humor abilities and verbal fluency measures. However, these associations were no longer significant within age groups, suggesting that they may primarily reflect age-related differences rather than direct relationships between fluency and humor abilities. These findings indicate that while humor engagement declines with age, the types of humor used and appreciated remain stable, emphasizing the relevance of humor for social interactions and potential interventions aimed at supporting wellbeing in older adults.

## 1 Introduction

### 1.1 Definition of humor

*What makes you instantly feel good?* Laughter is likely to be the behavioral manifestation of a well-known but poorly defined phenomenon: humor. Humor can be defined as a social and cognitive phenomenon that allows individuals to communicate, bond, and cope with their environment, eliciting positive emotional responses in both the sender and the receiver (Martin and Ford, 2018; Greengross, 2013). From a cognitive perspective, humor involves multiple processes related to its production and appreciation (Martin and Ford, 2018). Some researchers distinguish between self-beneficial humor and humor that is harmful for oneself (Saroglou and Scariot, 2002; Martin et al., 2003), also called light humor and dark humor (Hofmann et al., 2020). Importantly, humor is deeply embedded in social contexts. For instance, a recent study also suggests that social context plays an important role in humor: for example, humor is less common among older people living alone, probably due to the reduction in social interactions on which it depends (Heap et al., 2026). The literature agrees on the existence of several forms of humor (Martin and Ford, 2018), but few studies have shown interest in studying each form of humor, and no research has made a clear distinction between the different forms of humor.

### 1.2 Different forms of humor

Among the different forms of humor, in the present work, we focused on six forms that are most frequently considered in the literature: irony, puns, dark humor, parody, slapstick humor and satire (Taylor and Mazlack, 2004; Aillaud and Piolat, 2013; Obert et al., 2016; Martin and Ford, 2018).

*Irony*, defined as communicating the opposite of literal meaning (Obert et al., 2016), frequently elicits ambivalent or negative emotional responses contingent upon its target and delivery. *Puns* are jokes involving verbal play, where words sound similar but used in two different senses and are typically associated with a positive emotional valence (Taylor and Mazlack, 2004). *Dark humor* deals with serious situations such as death, illness, disability or war in an exaggerated way, beyond what is socially acceptable and is often characterized by a negative valence (Aillaud and Piolat, 2013; Martin and Ford, 2018). *Parody* is close to imitation, restoring only certain appearances, and is most often constructed from artistic creations such as songs or films (Baumgartner et al., 2012). Parody tends to induce a positively valanced response, especially when it invites shared cultural references. *Slapstick humor*—an absurd, nonsensical mode characterized by a grotesque atmosphere (Yankwich, 1955)—typically elicits immediate, physical laughter and a positively valanced response through its use of exaggerated situational effects. Finally, *satire* allows the expression of constructive social criticism through humor, highlighting the vices and abuses of society and individuals (Singh, 2012). It typically evokes complex emotional responses, mixing amusement with moral reflection, but often holds a negative valence.

### 1.3 Humor and cognition

Cognitive psychology views humor as a high-level cognitive process, and attempts to describe the mental processes involved in its appreciation and production (Martin and Ford, 2018). According to Suls’ (1972) incongruity-resolution model, humor appreciation involves a two-stage process: the detection of an incongruity and its subsequent resolution, which renders the stimulus understandable and humorous (Suls, 1972).

Based on this first cognitive modeling, various studies underlined the implication of close links between humor and high-level cognitive processes (Gold and Buckner, 2002). Indeed, detecting and resolving incongruities requires mental flexibility. Meaning change also requires retrieving active information from working memory (Shammi and Stuss, 2003). Various studies reported that people who had the most difficulty in choosing a humorous punchline also had poorer performance in working memory and mental flexibility (Uekermann et al., 2006). However, studies jointly evaluating the appreciation and production of humor in line with executive processes remain limited due to the lack of available and validated tools. For example, the Humor Styles Questionnaires (HSQ) is currently the only standardized questionnaire in French language (Saroglou and Scariot, 2002; Kazarian and Martin, 2004). It measures four styles of humor (affiliative, self-enhancing, aggressive, and self-defeating) but does not specifically assess the production and appreciation of different forms of humor.

While the incongruity-resolution model remains foundational, it has been criticized for neglecting the emotional dimension of humor, which becomes particularly relevant when examining older adults. More integrative models combine cognitive and emotional processes and suggest that humor also serves affective and motivational goals (Coulson and Kutas, 2001; Samson and Gross, 2012; Ruch, 2007).

### 1.4 Humor and aging

With aging, executive functions such as working memory, inhibition and cognitive flexibility tend to decline (Troyer et al., 1997; Salthouse, 2011). Chan et al. (2012) showed that semantic fluency predicts humor comprehension, highlighting the role of semantic retrieval and flexibility. These abilities are central to humor appreciation and production, and age-related decline can affect humor processing.

The first study on aging and humor reported that older individuals understood jokes less well but appreciated them more than younger participants (Schaier and Cicirelli, 1976). Similarly, older participants rated humorous materials as funnier (Shammi and Stuss, 2003). These findings suggest a dissociation between cognitive performance and emotional response: while humor processing may decline, emotional appreciation can remain stable or increase. Older adults experience difficulties with complex humor, but their ability to appreciate humor often remains intact (Martin and Kuiper, 1999). Studies agree that this decline affects humor-producing capacity, while emotional benefits persist (Martin and Ford, 2018).

Across adulthood, humor serves various psychological and social functions (Martin et al., 2003). Evidence suggests that older adults increasingly use humor as a tool for emotional regulation and to maintain positive social interactions. Socio-emotional selectivity theory (Carstensen, 1991) suggests that older adults prioritize positive emotions. This selective focus on positive affect can shape the way older adults engage with humor: they are more likely to appreciate forms of humor that enhance wellbeing, reinforce social connections, and promote emotional balance. This may explain why older people consider laughter an important factor for mood and tranquility, even when cognitive processing declines (Ruch et al., 2010).

### 1.5 Aims of the present study

Few studies have examined how aging affects the appreciation and production of specific humor types. Because humor is often treated globally, little is known about how different forms vary with age or emotional valence. This study investigates age-related differences in the appreciation and production of six humor forms—irony, parody, satire, dark humor, slapstick, and puns—using a new French questionnaire (Hevin et al., 2024).

We expect younger adults to obtain higher scores than older adults, reflecting age-related cognitive decline. Consistent with socio-emotional selectivity theory (Carstensen, 1991), we also predict a preference for positive humor (parody, puns, slapstick) over aggressive forms (dark humor, irony, satire), especially among older adults.

Based on incongruity-resolution models of humor processing, we anticipate that better cognitive functioning will be associated with greater humor appreciation. Finally, we examine the relationships between age, verbal fluency, and humor abilities.

## 2 Methods

### 2.1 Population

The study included 101 participants, divided into three age groups: the young adult group (*N =* 37, *M* = 25.8 years, *SD* = 3.99; range: 19–34), the middle-aged group (*N =* 27, *M* = 50.1 years, *SD* = 5.50; range: 40–59) and the older adult group (*N =* 37, *M* = 70.1 years, *SD* = 7.18; range: 61–83). Recruitment was conducted through announcements issued on social networks and on the campus of Picardie Jules Verne University (Amiens, France). All participants were native French speakers. None of them reported current or previous neurological illnesses, learning disabilities, or head trauma. In addition, the Montreal Cognitive Assessment (MoCA) was administered to all participants in order to confirm an efficient overall cognitive functioning (score at least equal to the norm, i.e., 26/30) (Nasreddine et al., 2005). The MoCA is a 30-point screening tool assessing multiple cognitive domains including attention, executive functions, memory, language, visuospatial abilities, and orientation. Participants’ characteristics are summarized in Table 1. All participants took part in this study on a voluntary basis, and gave their written consent after being provided with detailed information. This research was undertaken in accordance with the Declaration of Helsinki.

**Table 1:** Means (and standard deviations) of participant age, education level, socioeconomic index, and MoCA scores.

Gender	Young adults (*N =* 37)		Middle-aged (*N =* 27)		Old adults (*N =* 37)		Statistics	*p*-value
	Male (*N =* 20) 54.1%	Female (*N =* 17) 45.9%	Male (*N =* 9) 33.3%	Female (*N =* 18) 66.7%	Male (*N =* 17) 45.9%	Female (*N =* 20) 54.1%	*χ^2^*(2) *=* 2.71	*p =* 0.258
Age	25.8 (± 3.99)		50.1 (± 5.50)		70.1 (± 7.18)			
Level of education	6.54 (± 1.71)*		6.89 (±1.58)*		5.27 (± 2.09)		*χ^2^(2) =* 12.18	*p =* 0.002
Socioeconomic score	6.52 (± 1.39)*		6.54 (±1.41)*		5.57 (± 1.57)		*χ^2^(2) =* 9.90	*p* = 0.007
MoCA Score	28.3 (± 1.16)*		28.2 (±1.28)*		27.2 (± 1.20)		*χ^2^(2) =* 16.38	*p < 0*.001

Continuous variables are presented as means (± standard deviations). Group comparisons were conducted using Kruskal–Wallis tests. Gender distribution was compared using a chi-square test. Asterisks (*) denote significant differences compared to the Older Adults group (*p* < 0.05).

### 2.2 Materials

#### 2.2.1 The humor questionnaire

Although the present study was not designed as a full psychometric validation, these results provide initial support for the reliability of the instrument. Further research is needed to examine its factorial structure more thoroughly.

The humor questionnaire, detailed in Hevin et al. (2024), consists of 48 items assessing both appreciation and production of six forms of humor, three of which are generally considered to have a positive emotional valence (parody, slapstick, puns), and three a more negative valence (irony, satire, dark humor) (see Table 2 for an example for each form of humor) (Hevin et al., 2024). Each form is assessed with 8 items, half evaluating production and for other half appreciation. These items were presented randomly and half of them were rephrase in the negative form prior to analysis in order to avoid an acquiescence bias. For each item, participants were asked to indicate if the item corresponded to them (i.e., if they used or appreciated this style of humor) with a seven-point Likert-type scale from strongly disagree to strongly agree. For example, for satire, the participant had to indicate his/her level of agreement with the following items: *“Moral jokes about society make me laugh”* (appreciation dimension) and *“I don’t make moral jokes about society”* (production dimension).

**Table 2:** Example of items in the affirmative form for each form of humor for the appreciation dimension.

Forms of humour	Item in appreciation dimension
Irony	I laugh at jokes suggesting that it is sunny when it is raining.
Parody	Jokes involving imitations of famous people make laugh.
Satire	Moralistic jokes about society make me laugh.
Dark humor	Jokes about the poor make me laugh.
Slapstick	In front of a glass window, I have fun pretending to hit myself to make people laugh.
Puns	I laugh at jokes based on first and last names.

The questionnaire was developed *a priori* based on theoretical distinctions identified in the literature and was pre-tested (*N =* 56) to ensure its conceptual validity using a recognition task. In the present study, internal consistency was high (Cronbach’s *α* = 0.88), with coefficients ranging from 0.67 to 0.87 across the six humor dimensions. Although the present study was not designed as a full psychometric validation, these results provide initial support for the reliability of the instrument. Further research is needed to examine its factorial structure more thoroughly.

For each form of humor, scores were calculated separately for appreciation and production by averaging the corresponding items. Negatively worded items were reverse-coded prior to analysis, so that higher scores consistently reflected greater endorsement of the corresponding humor dimension.

This questionnaire was part of a larger research protocol assessing different cognitive functions. In particular, mental flexibility was explored using 2-min categorical and phonemic verbal fluency tests (Godefroy, 2008). Although these tasks are commonly used as indirect indicators of cognitive flexibility, they are known to be multidetermined, as they also rely on lexical access, phonological processing, and semantic memory. Therefore, verbal fluency reflects both executive and language-related processes. The number of correct responses for each test was retained for the analyses.

#### 2.2.2 Procedure

The research protocol was administered for 3 years from December 2021 to January 2024 at the university or at the participants’ homes. Each participant was seen individually, after giving his/her consent, the participant completed the humor questionnaire (between 15 and 20 min), and was then proposed the cognitive tests. The administration time for the entire assessment was on average 2 h.

#### 2.2.3 Data analysis

Statistical analyzes were performed using the Jamovi statistical software (version 2.2.5). The reported values are means and standard deviations. Partial eta-squared ( $\eta_{\text{partial}}^{2}$ ) are also reported and served as a measure of effect size. For all these analyses, the statistical level of significance was set at *α* = 0.05. For all the analyses, we checked the conditions of normality, homogeneity of variances and sphericity. To investigate the effect of age on the appreciation of different forms of humor, a mixed-design ANOVA was conducted to examine the interaction between the two factors: form of humor (irony, parody, satire, dark humor, slapstick, puns) and age. The same analyze was performed for the production of the different forms of humor. ANOVAs were followed by *post-hoc* comparisons between the young adults and old adults’ groups (Tukey’s honestly significant difference test). Even when the main effect was not significant, *post-hoc* comparisons were performed for exploratory purposes. Also, given the observed group differences in education level, an ANCOVA was conducted including education as a covariate. The main effect of age group remained significant, while the effect of education was not significant. Additional regression analyses including age and gender as predictors confirmed that age remained a significant predictor of humor scores, independent of gender. Similarly, further regression analyses that included socioeconomic status, educational attainment and the MoCA indicated that these were not significant predictors. To explore the links between humor and cognitive processes, we performed correlation analyses between the total scores for appreciation and production and categorical and phonemic verbal fluency scores.

## 3 Results

### 3.1 Appreciation of humor

**Figure 1:**
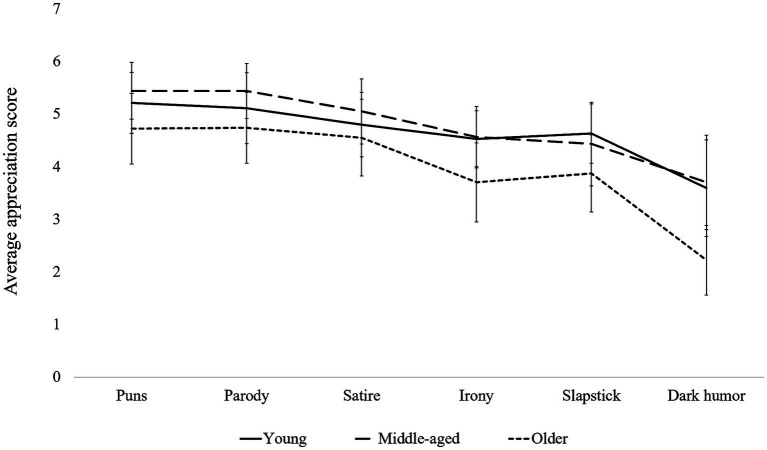
Average scores for appreciation by type of humor among young adults, middle-aged adults, and elderly people.

Regarding appreciation of humor, the ANOVA on the appreciation scores indicated a significant main group effect [*F*(2,98) = 7.82, *p* < 0.001, $\eta_{\text{partial}}^{2}$ = 0.138], a significant main effect of humor forms [*F*(5,490) = 43.01, *p* < 0.001, $\eta_{\text{partial}}^{2}$ = 0.305], but a non-significant Group × Humor forms interaction effect [*F*(5,490) = 1.55, *p* = 0.117, $\eta_{\text{partial}}^{2}$ = 0.031] (Figure 1).

*Post-hoc* analyses indicated that younger participants rated dark humor significantly lower than slapstick, parody, satire, and puns (all of *p* < 0.05). Similarly, participants in the intermediate age group reported lower appreciation for dark humor compared to satire, parody, and puns (all of *p* < 0.05). Among older adults, dark humor was also significantly less appreciated than slapstick, parody, satire, irony, and puns (all of *p* < 0.05). In addition, older adults rated irony lower than satire, parody, and puns (all of *p* < 0.05).

### 3.2 Production of humor

**Figure 2:**
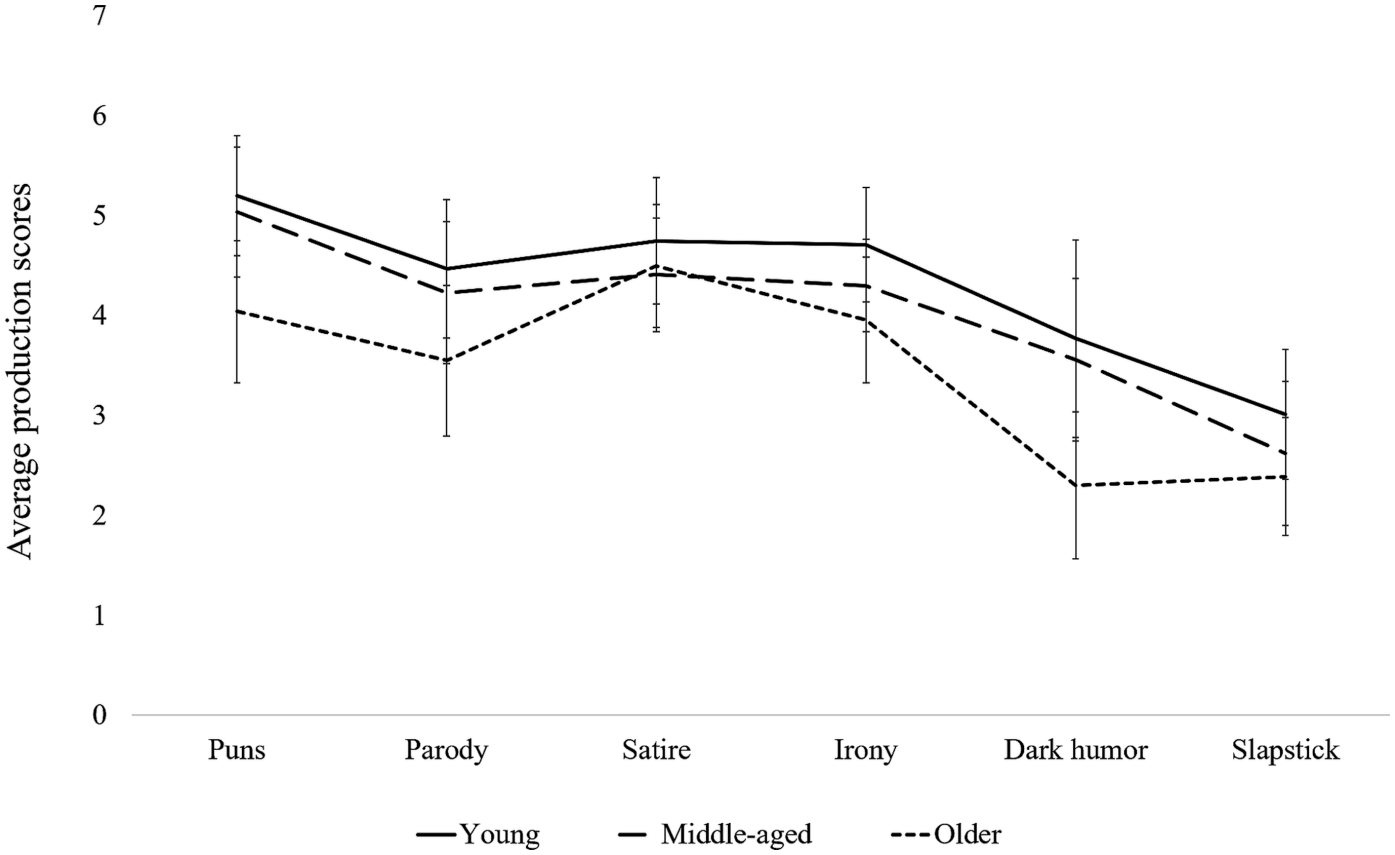
Average scores for production by type of humor among young adults, middle-aged adults, and elderly people.

The ANOVA on the production scores indicated a significant main group effect [*F*(2,98) = 8.64, *p* < 0.001, $\eta_{\text{partial}}^{2}$ = 0.15], a significant main effect of humor forms [*F*(5,490) = 52.95, *p* < 0.001, $\eta_{\text{partial}}^{2}$ = 0.351], and a significant Group x Humor forms interaction effect [*F*(5,490) = 2.04, *p* = 0.027, $\eta_{\text{partial}}^{2}$ = 0.04] (Figure 2).

Beyond the main group effect, young participants produced significantly more dark humor and puns than older adults (all *p* < 0.05). Production scores differed significantly across humor forms within each group. In the young adult’s group, the mean scores for slapstick humor were significantly lower than those for irony, satire, parody and puns (all *p* < 0.05). Moreover, in the young adult group, dark humor scores were significantly lower than those for satire and puns (respectively *p* = 0.02 and *p* < 0.05). For middle-aged participants, pun scores were significantly higher than those for slapstick humor and dark humor (all p < 0.05). In addition, slapstick humor scores were significantly lower than those for irony, satire, and parody (all *p* < 0.5). Finally, in the older adult group, dark humor and slapstick humor scores were significantly lower than all other humor forms (all *p* < 0.05). Furthermore, satire scores were significantly higher than parody scores (*p* < 0.05).

### 3.3 Correlation analyses

To explore the relationships between verbal fluency and humor-related abilities, we first conducted correlation analyses across the entire population. We observed significant positive correlations between the total appreciation score and categorical verbal fluency scores, *r* = 0.19, *p* = 0.03. Also, we observed a significant positive correlation between the total appreciation score and phonemic verbal fluency, *r* = 0.21, *p* = 0.02. With regard to humor production, there is a significant positive correlation between the overall production score and the categorical verbal fluency score, *r = 0*.23, *p =* 0.01. Conversely, production scores and phonemic fluency showed no significant correlation (*r* = 0.10, *p* = 0.15). No significant correlations were observed within individual age groups, suggesting that the associations found in the full sample are primarily driven by between-group differences (all of *p* > 0.05).

Finally, correlation analyses revealed that age was negatively associated with both humor appreciation and humor production scores (respectively, *r = −0*.36, *p* < 0.001 et *r =* −0.43*, p <* 0.05). Moreover, a negative correlation was also observed between age and categorical fluency (*r =* −0.27, *p* < 0.05).

## 4 Discussion

The present study was the first to examine age-related differences in the production and appreciation of six distinct forms of humor, using a self-report questionnaire. Our findings revealed that younger adults tend to produce and appreciate humor more than older adults. Although this pattern contrasts with some previous studies suggesting higher or more refined humor appreciation among older adults, the overall profiles of humor preferences remained largely similar across age groups, indicating a similar patterns of humor preferences across age groups. The relatively modest and unbalanced sample size may, however, have reduced statistical power—particularly for detecting interaction effects—warranting cautious interpretation of non-significant findings.

### 4.1 Humor appreciation and production across the lifespan

Our results indicate that younger adults reported higher scores for both humor appreciation and production than older adults, which is consistent with previous research (Shammi and Stuss, 2003; Mak and Carpenter, 2007; Daniluk and Borkowska, 2017). However, despite these overall quantitative differences, we found that the relative pattern of preferences across humor forms remained largely similar between age groups. This suggests that aging may affect the level of humor engagement while preserving preference structure.

The age-related declines in humor production varied by form: younger adults produced more puns and dark humor, whereas older adults reported significantly lower appreciation and production in slapstick and dark humor. This suggests that age-related changes in humor production may be partly explained by specific cognitive and socioemotional factors. According to the incongruity-resolution model (Suls, 1972), humor comprehension requires both detecting and resolving an incongruity. The observed decline in humor appreciation aligns with this model, as it may reflect a decrease in cognitive resources, particularly executive functions and flexible processing, involved in handling incongruity (Daniluk and Borkowska, 2017; Heap et al., 2026). This is particularly true for humor production, which is even more cognitively demanding than appreciation (Shammi and Stuss, 2003).

However, certain forms of humor, such as slapstick, rely more on the absurd than on the resolution of an incongruity, suggesting that socio-emotional factors may also explain differences between age groups. More broadly, these findings align with functional and emotion-regulation models of humor, suggesting that humor is not only a cognitive process but also a tool for managing emotions and social interactions (Martin et al., 2003). For instance, it has been suggested that younger adults often use humor to initiate social bonds, whereas older adults are more likely to employ it as a coping strategy (Martin et al., 2003; Wanzer et al., 2009). This functional shift could tentatively explain the lower reported humor production in older adults. Furthermore, social stereotypes associating old age with a lack of humor (Schmidt and Boland, 1986) might be internalized by older adults, thereby potentially affecting their self-reports (Levy, 2003; North and Fiske, 2013). Similarly, the reduced humor engagement in older adults living alone might highlight the importance of social context in humor use (Heap et al., 2026).

Regarding specific humor preferences, we expected older adults to show a stronger preference for positive humor forms, based on socioemotional selectivity theory, which posits that aging is associated with a motivational shift toward emotionally meaningful and positive experiences (Carstensen, 1991). Contrary to our hypothesis, non-aggressive forms of humor (i.e., puns, parody and slapstick humor) were not systematically the most appreciated and produced by older participants. Both younger and older adults more frequently favored puns, parody, and satire while slapstick and dark humor were consistently less favored. This pattern may indicate that socioemotional selectivity influences the regulation of emotionally negative humor rather than leading to a systematic preference for all positive forms. This selective reduction in dark humor among older adults may reflect age-related motivational changes, consistent with socioemotional selectivity theory, which emphasizes the prioritization of social harmony and emotional wellbeing (Carstensen, 1991). It is possible that older people are more prone to a social desirability bias (Stöber, 2001), perceiving aggressive humor as socially undesirable (Cann and Matson, 2014). Regarding the high use of satire among younger adults, one tentative interpretation is that this form of humor serves as a communication tool to incite behavioral change (Zekavat, 2019). Speculatively, in the context of the recent health crisis, satire would likely be employed to denigrate the decisions taken by governments and inappropriate behavior; such as lockdown restrictions or vaccination policies perceived as inconsistent, as well as to mock individuals who ignored social distancing or mask mandates (Zekavat, 2021).

Finally, consistent with previous research, men tend to report higher levels of aggressive humor than women (Hofmann et al., 2020). However, in the present study, gender differences did not account for the observed age-related effects. These preliminary results should be taken with caution as the size of our sample is the main limitation of this study. In addition, as the present study relies on a cross-sectional design, the observed differences reflect comparisons between age groups and should not be interpreted as within-individual developmental changes. In particular, cohort effects may have contributed to the observed patterns, as participants from different age groups may differ in their social, cultural, or historical experiences of humor. Future longitudinal studies are therefore needed to better disentangle age-related changes from cohort influences and to clarify how humor evolves over time.

### 4.2 The relationship between humor, verbal fluency, and aging

Our correlational analyses revealed associations between verbal fluency and humor abilities at the level of the full sample. However, these relationships were no longer significant when examined within age groups, suggesting that they may primarily reflect age-related differences rather than a direct link between fluency and humor abilities. In this context, the observed correlations may be driven by broader developmental or aging-related changes affecting both domains simultaneously, highlighting that these associations may reflect between-group differences rather than a stable relationship across the lifespan. However, alternative explanations such as reduced statistical power or restricted variability within groups cannot be ruled out.

Also, while humor appreciation and production appear to be associated with verbal fluency, this relationship likely reflects the combined influence of multiple cognitive processes, including linguistic abilities and executive functions, which are themselves modulated by age (Lezak et al., 2012). Verbal fluency tasks are multidetermined, and more refined indices such as clustering and switching may better capture cognitive flexibility (Troyer et al., 1997).

Given the exploratory nature of the study, verbal fluency was used as a global indicator. Future research should include finer-grained analyses (e.g., clustering and switching) to better disentangle the contributions of flexibility, language, and aging.

## 5 Conclusion

In conclusion, this study contributes to a more nuanced understanding of the relationship between age, cognition and humor by distinguishing six forms of humor across the dimensions of appreciation and production. While younger adults generally produce and appreciate humor more than older people, the favorite types of humor stay relatively consistent across age groups. Also, the findings suggest that verbal fluency is associated with humor abilities, reflecting both executive and language-related processes. Further research is needed to better disentangle the respective contributions of executive and linguistic processes, particularly in older adults. In the long run, further research could expand on these findings to explore how humor-based strategies can support healthy aging and enhance the overall wellbeing in older populations.

## Data Availability

The raw data supporting the conclusions of this article will be made available by the authors, without undue reservation.
